# Effects of Cryopreservation on Sperm with Cryodiluent in Viviparous Black Rockfish (*Sebastes schlegelii*)

**DOI:** 10.3390/ijms23063392

**Published:** 2022-03-21

**Authors:** Jingjing Niu, Xuliang Wang, Pingping Liu, Huaxiang Liu, Rui Li, Ziyi Li, Yan He, Jie Qi

**Affiliations:** 1 MOE Key Laboratory of Marine Genetics and Breeding, College of Marine Life Sciences, Ocean University of China, 5 Yushan Road, Qingdao 266003, China; niujingjing@ouc.edu.cn (J.N.); wangxuliang@stu.ouc.edu.cn (X.W.); liupingping@ouc.edu.cn (P.L.); liuhuaxiang@stu.ouc.edu.cn (H.L.); lirui2896@stu.ouc.edu.cn (R.L.); 21200611214@stu.ouc.edu.cn (Z.L.); yanhe@ouc.edu.cn (Y.H.); 2 Key Laboratory of Tropical Aquatic Germplasm of Hainan Province, Sanya Oceanog Institute, Ocean University of China, Sanya 572000, China

**Keywords:** black rockfish, sperm cryopreservation, sperm quality, transcriptome, methylome

## Abstract

Black rockfish is an economically important fish in East Asia. Little mention has been paid to the sperm cryopreservation in black rockfish. In this study, the optimal cryodiluent was selected from 48 combinations by detecting various sperm parameters. Transcriptome and methylome analysis were further performed to explore the molecular mechanism of inevitable cryoinjuries. The results showed that cryopreservation had negative effects on the viability, DNA integrity, mitochondrial activity, total ATPase and LDH of sperm even with optimal cryodiluent (FBS + 15% Gly). Transcriptome and methylome analysis revealed that the expression of 179 genes and methylation of 1266 genes were affected by cryopreservation. These genes were enriched in GO terms of death, G-protein coupled receptor signaling pathway, response to external stimulus and KEGG pathways of phospholipase D signaling pathway and xenobiotic and carbohydrate metabolism pathways. The role of PIK3CA and CCNA2 were highlighted in the protein-protein interaction network, and the sperm quality-related imprinted gene *mest* was identified among the 7 overlapping genes between transcriptome and methylome. Overall, the cryodiluent for black rockfish sperm was optimized, providing a feasible method for cryopreservation. The transcriptome and methylome data further demonstrated the underlying molecular mechanisms of cryoinjuries, proving clues for improvement of cryopreservation method of black rockfish.

## 1. Introduction

Black rockfish (*Sebastes schlegelii*), an important aquaculture species in China, Japan and Korea, displays the characteristics of viviparity and particularly long interval between copulation and fertilization. Unlike fish with external fertilization, male black rockfish transfer the sperm into the ovaries of females by mating in November or December [1,2,3]. The sperm can be stored to fertilize with mature oocytes in the ovary until the following March or April, and larvae are born one month after fertilization [1,2,3]. Wild resources of black rockfish have declined sharply in recent years as a result of commercial exploitation and environmental changes [4]. Artificial breeding and releasing become a crucial means of commercialization and resource supplementation. Sperm cryopreservation and artificial insemination have an important, positive impact on germplasm improvement of artificial breeding [5,6,7].

Sperm cryopreservation is a very useful teChinaique for reproductive management, conservation and genetic improvement of marine fish species. The program could synchronize the spermiation and ovulation during the breeding season, preserve high-quality sperm, simplify broodstock management and establish germplasm banks for endangered species [8,9]. More than 200 kinds of fish sperm cryopreservation protocols have been successfully established and reported, most of which were focused on endangered or economically important species [10].

The optimum method of cryopreservation varies among fish. The feasibility and effectiveness of each potential cryodiluent should be thoroughly investigated [11,12]. Successful cryopreservation of fish sperm is dependent on the selection of extender and cryoprotectant [8]. The extender is used as artificial seminal plasma to dilute sperm, and cryoprotectant is used to protect sperm against cryoinjuries during cryopreservation [13]. Several extenders and cryoprotectants have been used for different fish. For Atlantic salmon (*Salmo salar*), extender (Cortland’s medium) and cryoprotectant (1.3M dimethyl sulfoxide (DMSO) + 0.3M glucose (Gly) + 2% bovine serum albumin (BSA)) were used for sperm cryopreservation [14]. For sperm cryopreservation of rainbow trout (*Oncorhynchus mykiss*), Ginsburg fish Ringer as extender and MeOH as cryoprotectant were used [15]. Additionally, Mounib and propylene glycol (PG) were used as extender and cryoprotectant in sperm cryopreservation of winter flounder (*Pseudopleuronectes americanus*) [16]. 

Cryoinjuries are almost inevitable during sperm cryopreservation. The main cryoinjuries caused by cold shock and the formation of ice crystals can result in the oxidation of cellular compounds, disruption and damage of cellular structures, such as the DNA, acrosome and plasma membrane [17]. The expression of genes related to fertility was decreased in post-thaw human sperm, and proteins related to sperm motility, viability and acrosomal integrity were also significantly regulated by cryopreservation [18,19]. Additionally, hypermethylation of some genes was corroborated after cryopreservation [20]. Many of these genes were considered as male fertility markers, such as protamine 1 (*PRM1*, protamine 2 (*PRM2*) [21] and mesoderm specific transcript homolog (*MEST*) [22]. In mammals, *MEST* is associated with early embryo development and growth [23,24]. The expression and methylation of *MEST* could be affected, and its hypermethylation could lead to Silver–Russel syndrome in assisted reproductive teChinaology [25]. All the ultrastructural and molecular changes ultimately reduce post-thaw sperm quality.

At present, little attention has been paid to the sperm cryopreservation in black rockfish. It is a problem demanding a prompt solution to discover an available procedure for sperm cryopreservation of this economically important fish. In this study, we developed a cryodiluent for sperm cryopreservation of black rockfish by evaluating the sperm motility and kinematics via computer assisted sperm analysis (CASA) system. Accurate assessments of sperm viability, membrane functionality and mitochondrial activity was conducted by contemporary teChinaiques to evaluate the effects of cryopreservation on sperm. Transcriptome and methylome analysis were also performed to reveal the molecular changes of post-thaw sperm with cryoprotectant. The results contribute to establishing a method for sperm cryopreservation in black rockfish and providing clues for understanding the molecular mechanism of cryoinjuries and further improvement of cryopreservation of black rockfish.

## 2. Results

### 2.1. Optimization of Cryodiluent for Black Rockfish Sperm

**Figure 1:**
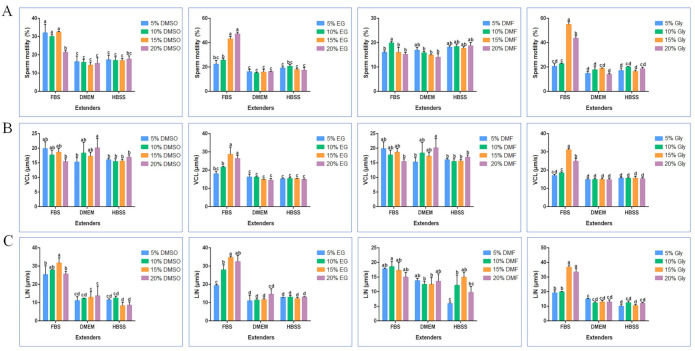
Effects of different cryodiluents on the post-thaw sperm motility parameters. (**A**) The motility of post-thaw sperm, (**B**) the VCL of post-thaw sperm, (**C**) the LIN of post-thaw sperm. VCL, curvilinear velocity; LIN (linearity, LIN = straight line velocity (VSL, µm/s) × VCL-1 × 100%). Statistical significance was accepted when *p* < 0.05 and indicated by different letters. FBS, fetal bovine serum; HBSS, Hank’s balanced salt solution; DMEM, Dulbecco’s modified eagle medium; DMSO, dimethyl sulfoxide; EG, ethylene glycol; DMF, *N*, *N*-dimethylformamide; Gly, glycerol.

In order to successfully cryopreserve black rockfish sperm and improved the motility of post-thaw sperm, the efficacy of 48 cryodiluents on sperm cryosurvival were evaluated. Significant differences (*p* < 0.05) were noted in fresh and post-thaw sperm and differences in the motilities of sperm cryopreserved with different cryodiluents were also noted (Figure 1A). Compared with the fresh sperm motility (83.2 ± 5.07%), the highest recovery of post-thaw sperm motility (55.09 ± 2.03%) was achieved when the sperm was cryopreserved with the cryodiluent (fetal bovine serum (FBS) + 15% glycerol (Gly)) (Figure 1A). No significant differences (*p* > 0.05) were shown in the curvilinear velocity (VCL) and linearity (LIN) of sperm cryopreserved with cryodiluent (FBS + 15% Gly) and fresh sperm (Figure 1B,C). The post-thaw motilities of sperm cryopreserved with FBS and 20% Gly, 15% or 20% ethylene glycol (EG) ranged from 43.03 ± 2.16% to 47.18 ± 1.46%, showing no significant differences (*p* > 0.05) (Figure 1A). When using N, N-Dimethylformamide (DMF) as a cryoprotectant, post thaw sperm motilities were less than 20% regardless of the extenders used. Fifteen percent Gly and FBS were found to be the optimal combination for the cryopreservation of black rockfish in 48 cryodiluents.

### 2.2. Cryopreservation Caused Sperm Quality Reduction

**Figure 2:**
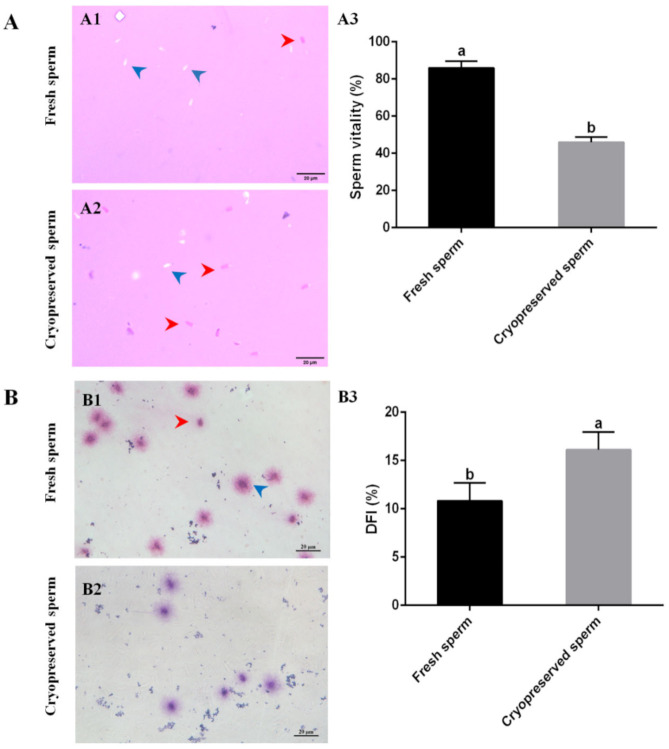
Effects of cryopreservation on sperm quality with the cryodiluent (FBS + 15% Gly). (**A**) Effects of cryopreservation on sperm plasma membrane integrity. (**A1**,**A2**) the eosin-aniline black staining. The dead sperm with damaged plasma membrane were stained to dark pink, whereas live sperm with plasma membrane integrity were not stained. (**A3**) Vitality of fresh and cryopreserved sperm. Sperm vitality measured as % of sperm without staining. (**B**) Effects of cryopreservation on sperm DNA integrity. (**B1**,**B2**) sperm chromatin dispersion (SCD) test. The sperm without fragmented DNA showed nucleoids with big halos of spreading of DNA loops, whereas those with fragmented DNA showed no halo or a small halo. (**B3**) DFI of fresh and cryopreserved sperm. DFI measured as % of sperm with no halo or a small halo. Scale bar = 20 µm. Statistical significance was accepted when *p* < 0.05 and indicated by different letters.

Cryopreservation resulted in significant reduction in post-thaw sperm motility even with the optimal cryodiluent (FBS + 15% Gly), suggesting that sperm quality was affected by cryopreservation. Sperm plasma membrane integrity (SPMI) and DNA integrity were used as two important parameters of sperm quality. The SPMI was examined by eosin-aniline black staining; in this way, the dead sperm with incomplete plasma membrane could be stained in dark pink by eosin (Figure 2A(A1 and A2)). An average of 85.98 ± 3.6% fresh sperm were not stained (Figure 2A(A3)). The stained sperm significantly (*p* < 0.05) increased after cryopreservation. Only 45.93 ± 2.7% of post-thaw sperm were not stained (Figure 2A(A3)). The sperm DNA integrity was determined by sperm chromatin dispersion (SCD) test; using this method, the sperm without fragmented DNA showed nucleoids with big halos of spreading of DNA loops. The results showed that most fresh sperm without fragmented DNA displayed a medium or large halo around the sperm head, but lots of cryopreserved sperm with fragmented DNA displayed no halo or a small halo (Figure 2B(B1,B2)). Further, the DNA fragmentation index (DFI) of cryopreserved sperm was significantly (*p* < 0.05) higher than that of fresh sperm (Figure 2B(B3)).

### 2.3. Deleterious Effects of Cryopreservation on Sperm Function

**Figure 3:**
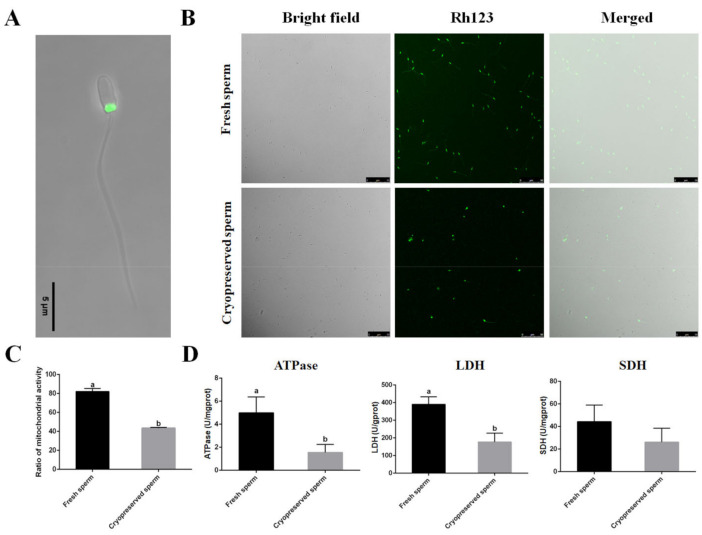
Effects of cryopreservation on sperm function with the cryodiluent (FBS + 15% Gly). (**A**) The location of mitochondria in black rockfish sperm. Scale bar = 5 µm. (**B**) Detection of mitochondrial membrane potential by Rh123. Scale bar = 50 µm. (**C**) Mitochondrial activity rate of fresh and cryopreserved sperm. Y-axis showed mitochondrial activity, measured as % of spermatozoa with green fluorescence in the mitochondrial area. (**D**) Detection of ATPase, LDH and SDH of fresh and cryopreserved sperm. Statistical significance was accepted when *p* < 0.05 and indicated by different letters.

The mitochondrion is a key organelle crucial for normal sperm functions. In black rockfish, mitochondria were found in the sperm’s midpiece (Figure 3A). The mitochondrial activity and enzyme activities related to energy metabolism were evaluated to assess the effects of cryopreservation on sperm function. Mitochondrial membrane potential was monitored by fluorescence of Rh123. The number of active mitochondria with green fluorescence was significantly (*p* < 0.05) decreased after cryopreservation, demonstrating that mitochondrial activity reduction occurred in the post-thaw sperm cryopreserved with the cryodiluent (FBS + 15% Gly) (Figure 3B,C). The activities of total ATPase, LDH and SDH were detected with Colorimetric Assay Kit. A significant (*p* < 0.05) decrease in ATPase and LDH activity was observed after cryopreservation, indicating that sperm metabolism was negatively regulated in the post-thaw sperm cryopreserved with the cryodiluent (FBS + 15% Gly) (Figure 3D).

### 2.4. Effects of Cryopreservation on Sperm Transcriptome

**Figure 4:**
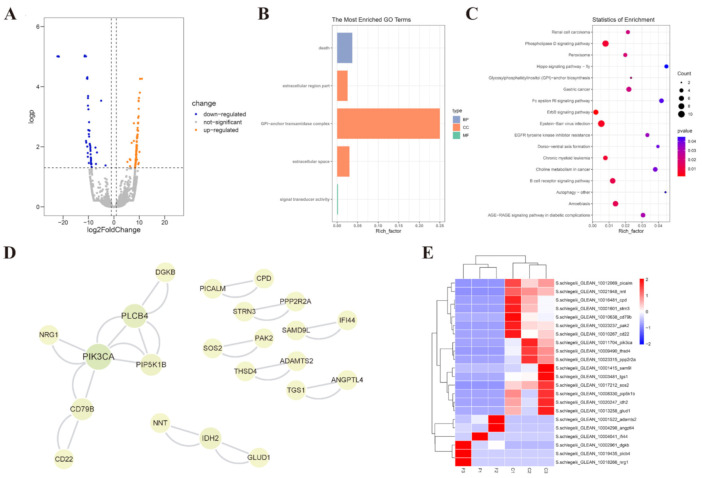
Transcriptome analysis of sperm with the cryodiluent (FBS + 15% Gly). (**A**) DEGs in transcriptome profile of cryopreserved sperm. One hundred seventy-nine DEGs were identified, including 97 upregulated genes and 82 downregulated genes. (**B**) GO enrichment analysis of DEGs. Statistical significance was accepted when *p* < 0.05. (**C**) KEGG enrichment analysis of DEGs. Statistical significance was accepted when *p*-value < 0.05. (**D**) The PPIN of DEGs. The PPIN was constructed by STRING database and visualized using Cytoscape software. The node size is positively correlated with degree. (**E**) The expression pattern of key DEGs identified in PPIN. The x-axis shows sampled tissues, with the prefix F for sperm without cryopreservation and C for sperm with cryopreservation, and the y-axis shows genes. The color scale showed standardized RPKM values normalized by Z-score method.

RNA sequencing was performed to obtain the transcriptomic profiles of fresh and post-thaw sperm cryopreserved with the cryodiluent (FBS + 15% Gly). Transcriptome analysis revealed 179 differentially expressed genes (DEGs), of which 82 and 97 genes were significantly downregulated and upregulated after cryopreservation, respectively (Figure 4A and Supplementary Data S1). GO analysis revealed that the DEGs were significantly (*p* < 0.05) enriched in five terms, including death, extracellular region part, extracellular space, GPI-anchor transamidase complex and signal transducer activity (Figure 4B and Supplementary Data S1). Except pathways related to diseases, the KEGG analysis revealed that these DEGs were significantly (*p* < 0.05) enriched in pathways involved in transport and catabolism, such as peroxisome and autophagy, and signal transduction, such as the phospholipase D signaling pathway (Figure 4C and Supplementary Data S1). Protein-protein interaction network (PPINs) were further constructed using all the DEGS and demonstrated that important function of phosphatidylinositol 4,5-bisphosphate 3-kinase catalytic subunit alpha (PIK3CA), whose encoding gene *pik3ca* was notably upregulated in cryopreserved sperm (Figure 4D,E and Supplementary Data S1).

### 2.5. Effects of Cryopreservation on Sperm Methylome

**Figure 5:**
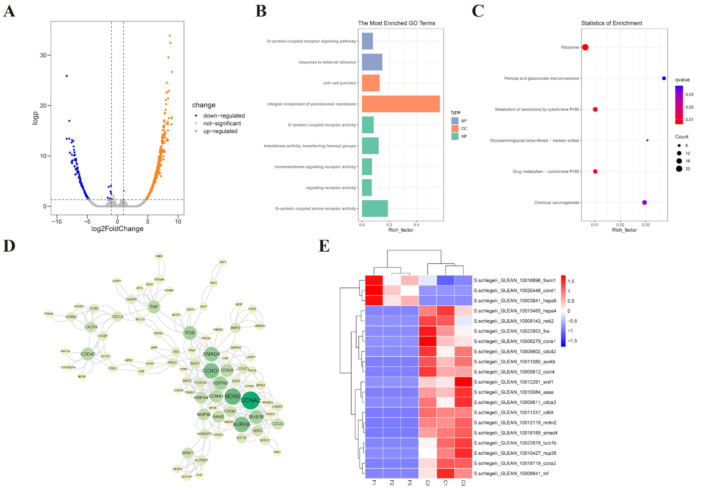
Methylation analysis of sperm with the cryodiluent (FBS + 15% Gly). (**A**) DMGs in the methylome profile of cryopreserved sperm. One thousand two hundred sixty-six DMGs were identified, including 261 downregulated genes and 1005 upregulated genes. (**B**) GO enrichment analysis of DMGs. Statistical significance was accepted when q-value < 0.05. (**C**) KEGG enrichment analysis of DMGs. Statistical significance was accepted when q-value < 0.05. (**D**) The PPIN of DMGs. The PPIN was constructed by STRING database and visualized using Cytoscape software. The node size is positively correlated with degree. (**E**) The expression pattern of key DMGs with a degree of 10 or above identified in PPIN. The x-axis showed sampled tissues, with the prefix F for sperm without cryopreservation and C for sperm with cryopreservation, and the y-axis shows genes. The color scale showed standardized RPKM values normalized by Z-score method.

Methylome profiles of fresh and post-thaw sperm cryopreserved with the cryodiluent (FBS + 15% Gly) were obtained to assess the effects of cryopreservation on sperm epigenetics. A total of 1266 differentially methylated genes (DMGs) were identified between the two sets, including 1005 upregulated and 261 downregulated DMGs (Figure 5A and Supplementary Data S2). Enrichment analysis showed that 9 GO terms were notably enriched, such as G-protein coupled receptor signaling pathway, response to external stimulus and integral component of peroxisomal membrane (Figure 5B and Supplementary Data S2). The KEGG enrichment analysis further revealed that the DMGs were enriched in pathways related to xenobiotic and carbohydrate metabolism, including metabolism of xenobiotics by cytochrome P450, drug metabolism-cytochrome P450, glycosaminoglycan biosynthesis-keratan sulfate and pentose and glucuronate interconversions (Figure 5C and Supplementary Data S2). The core PPIN further demonstrated the association of sperm cryoinjuries with the methylation of trophoblast development related gene cyclin-A2 (*ccna2*), which were significantly upregulated after cryopreservation (Figure 5D,E and Supplementary Data S2).

## 3. Discussion

The success of sperm cryopreservation of fish sperm can be influenced by many factors, including cryodiluent consisting of extender and cryoprotectant, equilibrium time, freezing rate and thawing temperature, of which the most important is cryodiluent [26,27]. In this study, the efficacy of cryodiluents developed from three extenders (FBS, HBSS, DMEM), and four cryoprotectants (DMSO, EG, DMF and Gly) at the concentrations of 5%, 10%, 15% and 20% on sperm cryopreservation of black rockfish were explored. The results showed that post-thaw sperm achieved the highest motility (55.09 ± 2.03%) by using the cryodiluent (FBS and 15% Gly). DMF as cryoprotectant resulted in low post-thaw sperm motility regardless of the concentration. 

Nearly 30% of post-thaw sperm were deemed immobile even by using the optimal cryodiluent (FBS and 15% Gly). The eosin-aniline black method was employed to assess the sperm plasma membrane integrity. The results showed that only 45.93 ± 2.7% of cryopreserved sperm were alive with complete plasma membrane, suggesting that death might be one reason for the immobility. Another reason for immobility was morphological defects, including abnormal head and broken tail (Figure S1). Post-thaw scanning electron microscopy also revealed loose sperm head and tail defects in Atlantic salmon [28]. Cryopreserved sperm concomitantly suffered more DNA damage as indicated by DNA fragmentation analysis, showing a significantly higher DFI. In fish, sperm with a high level of DNA damage presented low motility and velocity [29,30].

Other parameters were further detected to assess the effects of cryopreservation on sperm. Mitochondria are the energy source to maintain the physiological function of sperm. Rh123 was employed to detect the mitochondrial membrane potential, showing that mitochondrial activity significantly decreased in the cryopreserved sperm. Mitochondria supply energy for flagellar movement and their dysfunction could play a major part in structural and functional damage to the sperm [31]. Furthermore, a decrease in the activity of metabolism-related enzymes was also detected in cryopreserved sperm, suggesting that cell membranes integrity and mitochondria function of sperm could be damaged by cryopreservation. In fish and other vertebrates, LDH catalyzes the interconversion of pyruvate and lactate [32]. It was reported that lactate plus pyruvate seems to be the most favorable substrate to maintain ATP concentration and physiological level of adenylate energy charge of sperm from African catfish (*Clarias gariepinus*) during long-term storage at 4 °C [33]. In this study, the results showed that the activities of LDH were significantly reduced in the cryopreserved sperm, which were similar to the results in mandarin fish [34]. 

All these parameters, including sperm motility, viability, DNA integrity, MMP were negatively affected by cryopreservation even with cryodiluent, illustrating that cryoinjuries were almost inevitable during the sperm cryopreservation. The next generation sequencing has been employed to explore the molecular mechanism of cryoinjuries. In yellow catfish (*Pelteobagrus fulvidraco*), comparative transcriptome analysis revealed changes of gene expression in fresh and cryopreserved sperm and the effects of cryoprotectant Me_2_SO. Two thousand one hundred ninety-six DEGs, including 1214 upregulated and 982 downregulated genes, were identified when sperm was cryopreserved without Me_2_SO and enriched in the cAMP, PI3K-Akt and MAPK signaling pathways. The number of DEGs was remarkably decreased when Me_2_SO was used as cryoprotectant and only 110 DEGs containing 76 upregulated and 34 downregulated genes were identified [35]. In this study, only 179 DEGs were identified between non-cryopreserved sperm and cryopreserved sperm with cryodiluent (FBS + 15%Gly), the small amount of DEGs suggested a weak impact of cryopreservation on gene expression of sperm by using the method we established. However, the term of death, extracellular region part and space enriched in GO analysis also revealed the inevitable negative effects of cryopreservation on sperm survival and structure, which was consistent with the sperm parameters detected above. Among the 179 DEGs, the vital role of PIK3CA, which was significantly upregulated in post-thaw sperm, was highlighted in PPIN. PIK3CA is one important kinase of the catalytic subunit of PI3K, which plays a regulatory role in mammalian sperm physiology, such as motility, viability, capacitation and acrosome reaction [36,37,38,39]. The regulatory effect of the PI3K in sperm motility was also demonstrated in Atlantic croaker [40]. 

Recently, other studies have revealed the effects of cryopreservation on sperm DNA methylation, which is a major epigenetic modification and plays a crucial role in sperm nucleus compaction, gene silencing and prepatterning of embryonic gene expression [41]. For goldfish (*Carassius auratus*) sperm, global DNA methylation was not affected after cryopreservation with methanol. In contrast, cryopreservation with methanol induced a slight, but significant, increase in global DNA methylation of zebrafish (*Danio rerio*) sperm [41]. In addition, cryoprotectant could alter the pattern of sperm DNA methylation of tambaqui (*Colossoma macropomum*), decreasing the global methylation level [42]. There is little information about the effects of cryopreservation on the fish sperm methylome. In this study, 1266 DMGs were identified in methylome analysis, including 1005 significantly upregulated and 261 significantly downregulated genes. These DMGs were involved in GO terms of integral component of peroxisomal membrane. The peroxisome-related pathway and term were also enriched in KEGG analysis. Peroxisome is one source of reactive oxygen species (ROS) [43] and the impairment of ROS induced by duroquinone in sperm quality and hatching rate was demonstrated in common carp (*Cyprinus carpio*) [44]. To minimize the ROS-induced damages, various antioxidants have been added in extender during in vitro storage of fish sperm [45,46,47]. In this study, the pathway of peroxisome was also notably enriched in DEGs. These results suggested that cryopreservation might have an impact on the activity of peroxisome. Additionally, the association of sperm cryoinjuries with the methylation of CCNA2 was highlighted in core PPIN constructed using all DMGs. The methylation of *ccna2* gene in post-thaw sperm was upregulated in black rockfish. CCNA2 (Cyclin A2) is a member of the highly conserved cyclin family, involved in early vertebrate development [48,49,50]. In zebrafish, *ccna2* is one of the genes essential for embryonic and early larval development identified by a large insertional mutagenesis screen [50]. The essential role of CCNA2 in phosphorylation of UHRF1 (RING finger domains 1) during gastrulation was further revealed in *ccna2*^hi2696^ mutant zebrafish [51]. 

Furthermore, a total of 7 overlapping genes between DEGs and DMGs were identified, including dual specificity protein phosphatase 23 (*dus23*), B-cell lymphoma/leukemia 10 (*bcl10*), nuclear receptor coactivator 7 (*ncoa7*), autophagy-related protein 10 (*atg10*), mesoderm-specific transcript homolog protein (*mest*), microtubule interacting and transport domain containing 1 (*mitd1*) and NLR family card domain containing 3 (*nlrc3*), among which the key point is *mest*, an ortholog of mammalian imprinted gene *MEST* related to sperm quality [52]. In mammals, this gene is specifically expressed in the mesodermal tissue in the early stage of embryo [53], involved in fetal growth and development, placental function and postnatal behavior [54]. It has been repeatedly reported that hypermethylated *MEST* is associated with the male infertility phenotype [55,56,57,58]. *MEST* hypermethylation was also found in the sperm of male partners from couples experiencing recurrent pregnancy loss [59]. Cryopreservation could decrease the expression of *MEST* gene but not affect its DNA methylation pattern in human sperm [18,60]. In this study, the expression of *mest* was significantly downregulated, and the methylation of *mest* was significantly upregulated in post-thaw sperm, revealing that cryopreservation has an impact on sperm *mest* gene. Although the sperm quality was negatively affected by cryopreservation according the analysis above, the effects of cryopreservation on sperm fertility need to be further confirmed. However, internal fertilization and long interval between copulation and fertilization bring considerable trouble in fertility detection of cryopreserved sperm in black rockfish and more future researches is needed.

## 4. Materials and Methods

### 4.1. Sperm Collection

Three live males of black rockfish (body weight: 682 ± 49.0 g; total length: 35.1 ± 0.76 cm) were bought from Nanshan market of Qingdao, Shandong Province, China during the natural breeding season (November–December). Fish were anesthetized with 300 ppm tricaine methanesulfonate (MS-222; Sigma, Shanghai, China) before semen collection. For sperm collection, an abdominal incision was made to remove testis. Then, sperm in spermatic duct were squeezed out gently out and move into clean dry 2.0-mL cryogenic vials, and immediately placed on crushed ice until further use.

### 4.2. Cryopreservation and Thawing

The three-steps method was used with slight modification [61]. Semen was diluted with cryodiluent consisting of extender and cryoprotectant at 1:100 (*v*/*v*) ratio on crushed ice. Isopyknic diluted sperm samples from three fish were mixed, and a 500-μL aliquot of mixed sperm sample stored in a 2-mL cryogenic vial was incubated and equilibrated at refrigerator (4 °C) for 10 min. Then, the sperm sample was transferred to liquid nitrogen vapor (on the surface of liquid nitrogen) for 15 min. Finally, the sperm sample was stored in liquid nitrogen. Liquid nitrogen tank (MVE CryoSystem 750; MVE, Jinan, Shandong, China) was used for sperm cryopreservation.

In order to obtain the optimal cryodiluent, sperm (Number of fish = 3) was diluted in 48 cryodiluents developed from the combinations of three kinds of extenders (Fetal bovine serum (FBS), Hank’s balanced salt solution (HBSS), Dulbecco’s modified eagle medium (DMEM); BI, shanghai, China), and four kinds of cryoprotectants (dimethyl sulfoxide (DMSO), Ethylene glycol (EG), *N*,*N*-dimethylformamide (DMF) and glycerol (Gly); Solarbio, Beijing, China) and four different concentrations of cryoprotectants (5, 10, 15 and 20%). Sperm samples had been frozen in liquid nitrogen at least 48 h before thawing. The cryogenic vials were removed from the liquid nitrogen storage container carefully and immersed in a water bath (37 °C) for 40 s when thawed. Post-thaw sperm were placed on crushed ice and immediately used for motility analyses.

### 4.3. Sperm Motility Measurement

The sperm motility parameters, including sperm motility, curvilinear velocity (VCL, µm/s) and linearity (LIN = Straight line velocity (VSL, µm/s) × VCL-1 × 100%), were examined using computer-assisted sperm analysis (CASA) on a Sperm Class Analyzer (Microptic, Barcelona, Spain). Three microliters of fresh or post-thaw diluted sperm were immediately transferred to the sperm counting slide (SAS medical, Beijing, China), and recorded from 5 s to 30 s for measurement of sperm motility. Measurements were carried out in triplicate.

### 4.4. Sperm Viability Assessment

Sperm living solution (eosin-aniline black method) (Solarbio, Beijing, China) was used to examined sperm viability. First, a 20-µL semen sample was mixed with 20 µL of eosin staining solution and placed for 30 min at room temperature. Then, 60 µL of aniline black solution was added to the above mixture and placed for 30–60 s. Finally, one drop of the semen-eosin-aniline black solution was dripped on the slide. The viable sperm was white, and dead sperm was red under microscopic examination. At least 200 sperms were counted, and the percentage of living sperm was calculated as sperm viability.

### 4.5. Sperm DNA Integrity Testing

A sperm DNA fragment staining kit (Hezhong bioteChinaology, Sanming, China) was used for the SCD test. The fresh and cryopreserved sperm was measured according to the manufacturer’s protocol. The experiment was repeated three times and observed by microscopy AZ100 (Nikon, Tokyo, Japan). The DNA fragmentation index was further calculated based on the following formula.

DFI = (number of sperm without or with small halo)/(total count of sperm) × 100%

### 4.6. Mitochondrial Activity Detection

Mitochondrial membrane potential (MMP), a key indicator of mitochondrial activity, was detected by rhodamine123/Rh123 (Beyotime BioteChinaology, Shanghai, China) staining. Rh123 is a fluorescent cationic dye which preferentially penetrates the cell and enters into mitochondria based on highly negative MMP. Two hundred microliters of diluent with 2 × 10^6^ sperm was mixed with 1 µL of 0.25 µg/µL Rh123 and incubated in the dark for 10 min at room temperature. The stain results were observed by microscopy AZ100 (Nikon, Tokyo, Japan) and calculated for mitochondrial activity rate based on the following formula.

Mitochondrial activity rate = (number of sperm with fluorescence)/(total count of sperm) × 100%

### 4.7. Sperm Enzyme Activity Detection

Sperm was centrifuged at 600× *g* for 15 min at 4 °C to remove the seminal plasma or cryodiluent, and the bottom sperm was resuspended in 0.9% NaCl, then samples were stored at −20 °C for 24 h to lyse sperm. Finally, samples were centrifuged again at 600× *g* for 15 min at 4 °C and liquid supernatant was collected for enzyme activity assay. The enzyme activities of total adenosine triphosphatase (ATPase), succinate dehydrogenase (SDH) and lactate dehydrogenase (LDH) were detected by a colorimetric assay kit (Njjcbio, Nanjing, China) according to the manufacturer’s protocol.

### 4.8. Statistical Analysis

Data of sperm-related parameters represented mean values ± standard deviation (SD) of triplicate measurements. Significant differences among different data sets using the one-way ANOVA method in conjunction with Tukey’s multiple comparison post-test by SPSS statistics software version 20 (IBM Corp., Armonk, NY, USA). *p* < 0.05 was considered statistically significant.

### 4.9. Transcriptome Data

Total RNA of fresh and cryopreserved sperm was extracted with Trizol Reagent (Invitrogen, Carlsbad, CA, USA) according to instruction of the manufacturer. RNA quality was assessed using the Bioanalyzer 2100 system (Agilent TeChinaologies, Santa Clara, CA, USA). RNA-seq libraries were constructed and sequenced on the Illumina HiSeq platform (Tianjin Novogene Bioinformatics TeChinaology Co., Ltd., Tianjin, China). Averaged 46.93 Mb raw reads per sample were obtained in sperm RNA-seq. Averaged 45.05 Mb clean reads per sample were obtained by removing adapter sequences, empty reads and low-quality reads from raw reads by SOAPnuke [62]. The transcriptomic data were mapped to the black rockfish genome and the expression level of genes was calculated using Salmon [63] with default parameters. Genes showing an adjusted *p*-value < 0.05 and absolute Log2 fold-change (FC) ≥ 1 identified by DESeq were assigned as differentially expressed genes (DEGs). The volcano and heatmap were constructed by the ggplots and pheatmap package in R software (version 4.0.3). GO and KEGG enrichment analysis of DEGs were performed by using R script with *p*-value < 0.05. The DEGs were mapped to the STRING database (version 11.5) to construct a protein-protein interaction network (PPIN) and PPIN visualized by Cytoscape software [64].

### 4.10. Methylome Data

High-quality purified genome DNA was extracted by DNeasy Blood & Tissue kit (Qiagen, Shanghai, China). One hundred nanograms of DNA was digested by 5U restriction endonuclease FspEI (NEB, Beijing, China) at 37 °C for 4 h, the digested DNA product was verified by 1% agarose gel electrophoresis. The MethylRAD libraries were constructed as described by Wang et al. [65] and sequenced on a NovaSeq 6000 platform (Tianjin Novogene Bioinformatics TeChinaology Co., Ltd., Tianjin, China). Averaged 18.69 Mb raw reads per sample were obtained in MethylRAD-Seq. CG labels were extracted and mapped to the black rockfish genome (CNSA, ID CNA0000824). Differentially methylated genes (DMGs) were screened by bioconductor package edgeR [66] with absolute Log_2_ FC ≥ 1 and q-value < 0.05. The volcano and heatmap were constructed by the ggplots and pheatmap package in R software (version 4.0.3). GO and KEGG enrichment analysis of DEGs were performed by using R script with q-value < 0.05. The DEGs were mapped to STRING database (version 11.5) to construct a protein-protein interaction network (PPIN) and PPIN visualized by Cytoscape software [64].

## 5. Conclusions

In this study, we developed a cryodiluent for sperm cryopreservation in black rockfish. The post-thaw sperms achieved highest motility with the optimal cryodiluent (FBS + 15% Gly), but they also suffered inevitable cryoinjuries, including significantly decreased viability, DNA integrity and mitochondrial activity. The results could contribute to understating the molecular mechanism of cryoinjuries and provide clues for improvement of cryopreservation method for black rockfish.

## Data Availability

The datasets supporting the results of this article are included within additional files and available on request.

## Supplementary Materials

The following supporting information can be downloaded at: https://www.mdpi.com/article/10.3390/ijms23063392/s1.
