# Supplementary material for: Effects of Cryopreservation on Sperm with Cryodiluent in Viviparous Black Rockfish (Sebastes schlegelii)
Source: Int J Mol Sci. 2022 Mar 21;23(6):3392. doi: 10.3390/ijms23063392 (PMC8955014; doi:10.3390/ijms23063392)
Supplement: Supplementary file 1 [file ijms-23-03392-s001.zip › Figure S1.pdf]

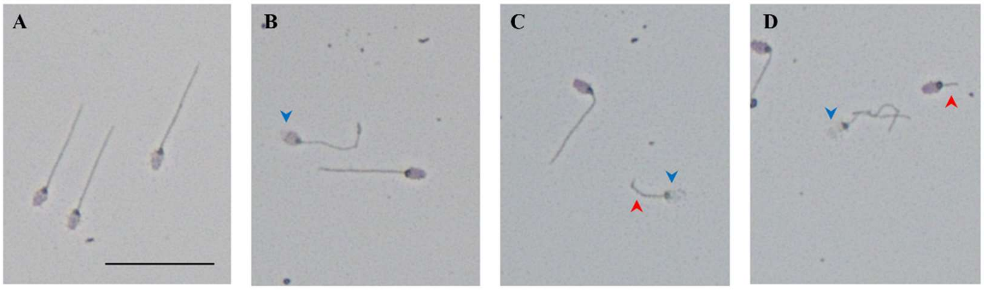

Figure S1. The morphology of fresh and post-thaw sperm. (**A**) Morphology of fresh sperm. (**B**–**D**) Morphology of post-thaw sperm. The sperm was dyed by sperm morphology rapid staining solution (Solarbio, Beijing, CHN). Abnormal head, indicated by blue arrowhead; broken tail, indicated by red arrowhead. Scale bar = 20 μm.
